# Presentations due to acute toxicity of psychoactive substances in an urban emergency department in Switzerland: a case series

**DOI:** 10.1186/s40360-016-0068-7

**Published:** 2016-05-26

**Authors:** Evangelia Liakoni, Patrick C. Dolder, Katharina M. Rentsch, Matthias E. Liechti

**Affiliations:** Division of Clinical Pharmacology and Toxicology, University Hospital Basel, Hebelstrasse 2, CH-4031 Basel, Switzerland; Laboratory Medicine, University Hospital Basel and University of Basel, Basel, Switzerland

**Keywords:** Recreational drugs, Acute toxicity, Psychoactive substances

## Abstract

**Background:**

Although the recreational use of psychoactive substances is common there is only limited systematic collection of data on acute drug toxicity or hospital presentations, in particular regarding novel psychoactive substances (NPS) that have emerged on the illicit market in the last years.

**Methods:**

We included all cases presenting at the emergency department (ED) of the University Hospital of Basel, Switzerland, between October 2014 and September 2015 with acute toxicity due to self-reported recreational drug use or with symptoms/signs consistent with acute toxicity. Intoxications were confirmed using immunoassays and LC-MS/MS, detecting also novel psychoactive substances.

**Results:**

Among the 50’624 attendances at the ED, 210 were directly related to acute toxicity of recreational drugs. The mean patient age was 33 years and 73 % were male. Analytical drug confirmation was available in 136 cases. Most presentations were reportedly related to cocaine (33 %), cannabis (32 %), and heroin (14 %). The most commonly analytically detected substances were cannabis (33 %), cocaine (27 %), and opioids excluding methadone (19 %). There were only two NPS cases; a severe intoxication with paramethoxymethamphetamine (PMMA) in combination with other substances and an intoxication of minor severity with 2,5-dimethoxy-4-propylphenethylamine (2C-P). The most frequent symptoms were tachycardia (28 %), anxiety (23 %), nausea or vomiting (18 %), and agitation (17 %). Severe complications included two fatalities, two acute myocardial infarctions, seizures (13 cases), and psychosis (six cases). Most patients (76 %) were discharged home, 10 % were admitted to intensive care, and 2 % were referred to psychiatric care.

**Conclusion:**

Most medical problems related to illicit drugs concerned cocaine and cannabis and mainly included sympathomimetic toxicity and/or psychiatric disorders confirming data from the prior year. Importantly, despite the dramatic increase in various NPS being detected in the last years, these substances were infrequently associated with ED presentations compared with classic recreational drugs.

## Background

The recreational use of psychoactive substances is common. It is estimated that over 80 million adults, or almost a quarter of the adult population in the European Union have tried illicit drugs at some point in their lives. The most commonly used drugs are cannabis, cocaine, amphetamines and 3,4-methylenedioxy-methamphetamine (MDMA), however, levels of lifetime use differ considerably between countries [1]. Collection of substance use-related data is usually based on indicators such as custom seizures, drug-related deaths, and surveys, but there is limited systematic collection of data on acute drug toxicity or hospital presentations. Moreover, novel psychoactive substances (NPS) have emerged in the last years in response to market trends and legislative control [2]. These novel substances (also known as “designer drugs”, “research chemicals”, “bath salts”, “plant food”, or “legal highs”) are usually analogues or derivatives of controlled substances, produced in order to circumvent regulations and imitate the effects of the controlled drugs. The number of NPS on Europe’s drug market has rapidly increased over the last decade. In 2014, 101 NPS were detected for the first time, 81 in 2013 [1]. The novel substances are typically not detectable with the usual drug of abuse immunoassays. It is therefore possible that they can contribute to acute toxicities and medical complications, or even deaths and escape detection. Good organized monitoring systems for drug-related health emergencies can help to expand the limited data currently available on acute toxicities of recreational substances in general and on NPS in particular, and thus contribute to the prevention of medical emergencies and to the improvement of the management of acute drug toxicity [3, 4].

The present study aimed to describe the acute recreational drug toxicity resulting in attendances to a large urban emergency department (ED) in Switzerland over the period of 1 year. Data on demographics, clinical findings, substances used, and short-term outcome of patients were collected. Additionally, the study specifically screened for NPS using liquid chromatography–mass spectrometry/mass spectrometry (LC-MS/MS). The study was performed for a second year in a row, continuing our previous study [5]. The study centre is part of the European Drug Emergencies Network (Euro-DEN), a 2-year project which has set up a network of sentinel centres across Europe to collect systematic data on presentations to the ED with acute drug/NPS toxicity [3, 6].

## Methods

This study was approved by the local ethics committee (Ethikkommission Nordwest- und Zentraschweiz (EKNZ), Basel, Switzerland). We included all patients admitted to the ED of the University Hospital of Basel with acute recreational drug-related medical problems between October 1^st^, 2014 and September 30^th^, 2015. The ED is both a primary care facility (walk-in patients) and a tertiary referral centre for hospitals in the greater Basel area. Additionally, all patients brought by paramedics are first admitted to the ED.

Cases were retrieved in monthly intervals from the electronic patient chart data-base using a comprehensive full-text search algorithm. In brief, the sensitive automatic search identified all cases with notions of abuse, intoxication or related terms and a large number of substance names including abbreviations and misspellings. The charts of all cases were reviewed, including notes by paramedics. Only patients with acute toxicity were included. A recreational drug was defined as “a psychoactive compound that was taken for the purpose of recreational activities rather than for medical or work purposes or for self-harm”. We identified the recreational drug(s) associated with the presentation based on one or a combination of the following: the patient’s self-reported use, information retrieved from witnesses, the opinion of the physician assessing the patient, and/or the analytical confirmation. Cases in which no information was available by the patient (e.g. because of coma at presentation, unwillingness to cooperate, language problems) but the symptoms and – if available- the analytical test were typical for an acute recreational drug toxicity, were also included. Cases with positive analytical tests without clinical signs of acute toxicity were not included. Data abstracting was performed in a standardized manner [3, 6] by one of the authors for the entire study. Data were collected within the Euro-DEN project [3]. Exclusion criteria were: Isolated ethanol intoxication (i.e. self-report of alcohol use only without analytical evidence of psychoactive substance co-use, where available), drug withdrawal and secondary complications of chronic drug use (e.g. infected injection sites).

We recorded the patient demographics (age, sex, hour and week day of ED admission), the substances used as reported by the patient or witnesses, the clinical effects, and clinical outcome. Clinical variables included the Glasgow Coma Scale (GCS) score, heart rate, blood pressure, respiratory rate, body temperature, laboratory tests and electrocardiography (ECG) findings. Hyperthermia was defined as a temperature >39 °C, hypertension as a systolic blood pressure ≥180 mmHg, hypotension as a systolic blood pressure ≤90 mmHg. Hallucinations were defined as any false or altered perception (visual, auditory, tactile, olfactory or gustatory), psychosis as any episode of delusions accompanied by confusion, hallucinations and lack of insight. The severity of poisoning was assessed using the Poison Severity Score for grading acute poisoning [7]. Mild toxicity refers to mild, transient and spontaneously resolving symptoms, moderate toxicity refers to pronounced or prolonged symptoms, and severe toxicity indicates severe or life-threatening symptoms.

CEDIA immunoassays (Thermo Fisher Scientific, Passau, Germany) were used to screen for barbiturates, amphetamines (including MDMA), benzodiazepines, cocaine, cannabis, methadone, and heroin. DRI immunoassays (Thermo Fisher Scientific, Passau, Germany) were used to screen for tricyclic antidepressants and opiates. Ethanol blood levels were determined by an enzyme assay. Additionally, LC-MS/MS analysis with a method covering over 770 substances was applied for confirmation and to detect additional substances in 78 of the cases (37 %) [8]. Levels of γ-hydroxybutyrate (GHB) were determined by an enzymatic assay (Bühlmann, Allschwil, Switzerland).

## Results

During the study period there were 50′624 emergency department attendances of which 210 were directly related to acute toxicity of drugs of abuse and therefore included in the present study. The demographic data are presented in Table 1. The mean patient age was 33 years and most of them were male (73 %). Most patients were admitted to the ED at night and/or on weekends, and half of them (52 %) were brought to the ED by ambulance.

**Table 1:** Patient characteristics

	Number of cases, *N* = 210 (%)
Male	154 (73)
Female	56 (27)
Age (years)	
≤20	36 (17)
21–30	64 (30)
31–40	57 (27)
>40	53 (25)
Time of presentation	
Night arrival (20:00 – 8:00 h)	107 (51)
Weekend arrival (Friday 17:00 h – Monday 8:00 h)	109 (52)
Ethanol co-ingested (self-reported)	
Yes	101 (48)
No	12 (6)
Not known	97 (46)
Self-reported drug use	
1 substance	146 (70)
>1 substances	51 (24)
No drug use	3 (1)
No information available (e.g. coma, incooperative)	10 (5)
Laboratory-confirmed drug use	
1 substance	55 (26)
>1 substances	72 (34)
Negative test (absence or insufficient test sensitivity)	9 (4)
No drug test performed	74 (35)

**Fig. 1:**
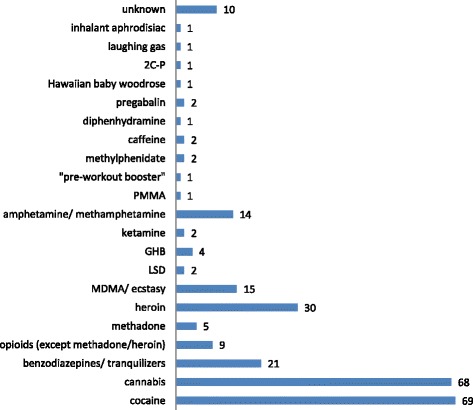
Self-reported substance use (count of cases)

The most commonly self-reported recreational drugs were cocaine and cannabis (Fig. 1). There were only 2 cases of relatively novel substances: A severe intoxication (GCS 3 and hypertension) with paramethoxymethamphetamine (PMMA) in combination with other substances (MDMA, methadone, benzodiazepines) and an intoxication of minor severity (GCS 14 and dizziness) with 2,5-dimethoxy-4-propylphenethylamine (2C-P), both self-reported without analytical confirmation. Ten patients (5 %) reported that they have used a substance without knowing what it was. In 10 cases (5 %) there was no information available on the agents taken and three patients (1 %) denied having used any drugs. These patients were included because they were judged by the assessing physician as being acutely intoxicated based on the symptoms and/or analytical confirmation. Seventy percent of the patients reported use of only one substance, while use of more than one recreational drug was reported in 24 % of the cases. Co-use of alcohol was reported in 48 % of the cases (Table 1).

**Fig. 2:**
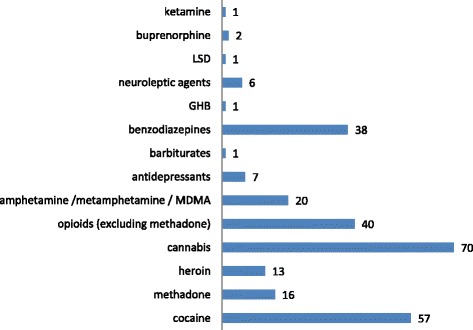
Substances analytically confirmed (count of cases)

An analytical drug confirmation was available in 65 % of the total cases (immunoassay in 136 cases, additional LC-MS/MS in 78 cases). The most commonly analytically detected substances were cannabis and cocaine, followed by opioids (excluding methadone) and benzodiazepines (Fig. 2). In 34 % of the cases more than one substance was analytically confirmed (Table 1).

Table 2 summarizes the medical problems. The most commonly reported symptoms were tachycardia (28 %), anxiety (23 %), nausea or vomiting (18 %), and agitation (17 %). One hundred-two patients (49 %) had an impaired consciousness (GCS <15), among them 43 were unconscious (GCS <8) at presentation or pre-hospital. Among all 210 cases 46 (22 %) presented with severe intoxication and there were two fatalities (one case with MDMA and one case with heroin) (Table 3). In 28 of the 46 severe intoxication cases concomitant use of alcohol was reported and/or analytically confirmed. Severe complications further included two acute myocardial infarctions (both after use of cocaine, in one case in combination with crystal meth), seizures (13 cases), and psychosis (six cases) (Table 2). Most patients (76 %) were medically discharged after spending less than 24 h at the ED, in 17 cases (8 %) the patients took their own discharge or left the ED before being seen by a doctor. Twenty patients (10 %) were admitted to the critical care unit, in nine cases (4 %) the patients were hospitalized in wards other than critical care unit, and four patients (2 %) were admitted to a psychiatric clinic (Table 3). In 152 cases (72 %) medical treatment including oxygen and intravenous fluid administration was provided. Tracheal intubation was performed in seven cases (3 %). Sedating drugs (e.g. benzodiazepines, antipsychotics, propofol, ketamine) were administered in 50 cases (24 %), an antidote (i.e. naloxone, flumazenil, and/or biperiden) was given in 18 cases (9 %).

**Table 2:** Clinical characteristics of acute recreational drug intoxications

	Number of cases, *N* = 210 (%)
Cardiovascular	
Chest pain	32 (15)
Palpitations	26 (12)
Dyspnea	18 (9)
Hypertension (systolic blood pressure ≥180 mmHg)	5 (2)
Hypotension (systolic blood pressure ≤90 mmHg)	3 (1)
Tachycardia (>100 beats per minute)	58 (28)
Myocardial infarction	2 (1)
Arrhythmias	2 (1)
QTc >450 msec	32 (15)
Psychiatric	
Anxiety, nervousness, or fear	49 (23)
Psychosis	6 (3)
Hallucinations	8 (4)
Agitation or aggression	35 (17)
Panic attack	8 (4)
Insomnia	5 (2)
Suicide ideation	1 (<1)
Neurologic	
Unconscious (GCS <8) at presentation or pre-hospital	43 (20)
Impaired consciousness (GCS <15) at presentation or pre-hospital	102 (49)
Vertigo or dizziness	16 (8)
Headache	6 (3)
Paresthesias	6 (3)
Seizure	13 (6)
Tremor	5 (2)
Amnesia	7 (3)
Cerebellar features (e.g. ataxia)	3 (1)
Miosis	16 (8)
Mydriasis	6 (3)
Respiratory depression	7 (3)
Miscellanous	
Hyperventilation	12 (6)
Nausea or vomiting	38 (18)
Diarrhoea	1 (<1)
Sweating	8 (4)
Malaise	6 (3)
Abdominal pain	11 (5)
Hyperthermia >39.0 °C	1 (<1)
Muscle cramps	1 (<1)
Pneumothorax	1 (<1)
Injuries (e.g. fracture, wound)	1 (<1)
Epistaxis	2 (1)
Elevated creatine kinase (>250 U/L)	59 (28)
Weakness, walking impairment	2 (1)

**Table 3:** Severity of poisoning and outcome

	Number of cases, *N* = 210 (%)
Severity of poisoning	
Minor	119 (57)
Moderate	43 (20)
Severe	46 (22)
Fatal	2 (1)
Outcome	
Medically discharged home	160 (76)
Self-discharged	17 (8)
Admission to critical care unit	20 (10)
Admission to ward other than critical care unit	9 (4)
Admission to psychiatric clinic	4 (2)

## Discussion

In this study we described acute medical problems due to recreational drug use. Cocaine and cannabis were the substances most commonly self-reported and analytically detected, while only 2 ED presentations were related to acute toxicity of NPS including 2C-P and PMMA. The severity of the intoxication was minor in the majority of the cases. However, there were two fatalities, one case with MDMA and one case with heroin. Most medical problems included sympathomimetic toxicity and/or psychiatric disorders. Ethanol co-use was reported in almost half of the cases and in the majority of the severe intoxications alcohol co-use was reported and/or analytically confirmed.

In comparison to the data from the same ED from the prior year (October 1^st^, 2013 - September 30^th^, 2014) [5] there was a similar number of cases (210 in the present study, 216 in the prior year). The substances most commonly associated with presentations to the ED due to acute drug toxicity were in accordance with the prior year cocaine and cannabis. There were two presentations due to NPS acute toxicity in both studies, however, different NPS were reported each year (delusional parasitosis under pentylone and hallucinations under 2,5-dimethoxy-4-bromophenethylamine (2C-B) in the prior year). Regarding the chemical structure, all four NPS seen in both our studies (PMMA, 2C-P, 2C-B, and pentylone) were phenethylamines, a large family of monoamine alkaloids which also includes amphetamine, methamphetamine, and MDMA [2, 9]. Phenethylamine is the core structure of many NPS, but little modification can lead to significant alterations in pharmacological properties and clinical actions [2, 9, 10]. PMMA, often sold as MDMA or in combination with MDMA in ecstasy pills, has been associated with severe hyperthermia [2, 10, 11]. In the PMMA case in our study, the 42-year old male patient reported having used “superman-XTC” tablets containing MDMA and PMMA, in combination with methadone and diazepam. Symptoms included tachycardia (117 beats per minute at presentation), hypertension (systolic blood pressure 197 mm Hg at presentation), and a GCS of three pre-hospital (14 at presentation), but the body temperature values were not reported. Because of the multiple substances used the clinical toxicity may not have been caused by PMMA alone in this case. 2C-P is a member of the 2C-series of substituted phenethylamines, a large family of hallucinogens characterized by methoxy-groups at positions 2 and 5 of the benzene ring and different hydrophobic substitutions at the 4-position [2, 9, 10]. The case in our study was a 22-year old male patient who reported dizziness after consuming alcohol (3 l of beer) together with a tablet initially thought to contain MDMA but which later turned out to contain 2C-P. Because of the co-use of alcohol the reported dizziness and GCS score of 14 cannot be attributed to the 2C-P toxicity alone, if at all.

Similar to the previous year, medical problems mainly included sympathomimetic toxicity and/or psychiatric disorders, and most patients could be discharged home. Severe toxicity was in both studies more frequent in combination with ethanol use, indicating that factors which contribute to the characterization of an intoxication as “severe” (e.g. impaired consciousness) could be in many cases due to alcohol use and not directly related to the other recreational substances. Additionally, in 13 cases (46 %) of the severe intoxications with alcohol co-use (*n* = 28) other CNS depressants (e.g. benzodiazepines, opioids, GHB) were self-reported and/or analytically confirmed in contrast to only seven cases (25 %) with co-use of stimulants (e.g. cocaine).

Compared to the prior year there was an increase in the cases related to heroin (30 vs. 15 self-reported cases), and a decrease in the cases related to LSD (2 vs. 11 self-reported cases). However, because of the small sample size and short period of observation, these trends may have occurred by chance.

In 16 EDs across Europe the most often reported drugs used during 1 year (October 1^st^, 2013 - September 30^th^, 2014) were heroin, cocaine, and cannabis [6]. Similar to our findings in Basel, NPS were involved in only 5.6 % (*n* = 484) of the cases (mainly mephedrone). Together the data indicates that NPS are either consumed to a lesser extent compared to classical recreational drugs, and/or show less acute toxicity.

One of the reasons for providing systematic collection of data on acute psychoactive substance toxicity is to improve recognition and clinical management of drug related emergencies. Treatment includes in most of the cases general supportive measures, as for most of the substances there is no specific antidote available. Specific therapy is available only for few substances, such as opioids (naloxone) and benzodiazepines (flumazenil). The opioid antagonist naloxone can reverse the effects of most opioids within minutes, which may be life-saving in the case of a massive overdose [12]. In cases of co-use of opioids with cocaine though, the treatment with naloxone may lead to life threatening arrhythmias [13]. Although in the present study naloxone was given in six cases of opioid co-use with cocaine (self-reported and/or analytically detected), no such complications were reported. In one of the fatality cases in our study, the 19-year old patient was brought to ED from a night club with impaired consciousness (GCS 12–13), tachycardia (180 beats/min), cramps, and foam in his mouth, after consuming 3 or 4 green pills in diamond shape with a superman logo (reportedly MDMA and confirmed later with LC-MS/MS). Shortly after admission, it came to a rapid deterioration with the GCS dropping to five, hyperthermia (>42 °C), hypertension, and tachycardia, initially with a wide complex tachycardia which soon turned into ventricular fibrillation and finally asystole and exitus despite reanimation. Intoxication with MDMA can lead to severe hyperthermia. It is possible that timely measures to reduce the temperature may have led to another outcome in this case. Temperature can be reduced in such cases by removing clothing, spraying with tepid water, and encouraging evaporative cooling with fanning. For very high body temperatures (>40–41 °C), neuromuscular paralysis is used to abolish muscle activity quickly [12]. Benzodiazepines (e.g. diazepam 0.1–0.3 mg/kg per os, intravenously, or per rectum) are recommended in the treatment of acute MDMA toxicity to suppress CNS sympathetic outflow [14]. In our case the patient received midazolam before intubation.

Among the substances used for recreational purposes in our study were also prescription medicines and other products, not typically associated with recreational use (Fig. 1). The recreational misuse of prescription drugs in Europe is an increasing issue of concern. Sedatives and hypnotics are after opioids the most commonly misused prescription drugs [15]. Benzodiazepines were the most common group of recreationally used prescription drugs in a European study [6]. Reasons for their misuse reported in surveys included insomnia, stress, and/or to get high [16]. Pregabalin was also commonly reported across Europe [6]. Pregabalin may have effects on the dopaminergic system [17] and a potential for misuse [18].

Our study has some limitations. Substances that have been taken as co-medication (e.g. antidepressants), given as a treatment by the paramedics (e.g. benzodiazepines), or can be detected in samples beyond the acute intoxication (e.g. cannabis) could have been overrepresented in the analytical results. On the other hand, substances like GHB, which can be detected only during a very short time period after use, and synthetic cannabinoids, which cannot be detected with the LC-MS/MS method used in the present study, may have gone undetected. Furthermore, there were some missing data in the patient histories, clinical data were not recorded in standardized manner at presentation, some symptoms described could have been the result of withdrawal rather than acute toxicity (e.g. seizures associated with alcohol and/or benzodiazepine withdrawal), and data from only one ED is not representative, as it may reflect local trends.

Strengths of our study include the detailed patient and clinical data documentations used, in contrast to studies based on coded diagnoses or analyses of poison center data. Especially, the exposure was confirmed for the majority of the patients.

## Conclusion

In conclusion, most medical problems related to recreational drug toxicity concerned cocaine and cannabis and mainly included sympathomimetic toxicity and/or psychiatric disorders. Similar to the previous year, cases with acute toxicity linked to NPS appear to be uncommon.
